# Prostatic displacement during extreme hypofractionated radiotherapy using volumetric modulated arc therapy (VMAT)

**DOI:** 10.1186/s13014-014-0262-y

**Published:** 2014-11-28

**Authors:** Adam Gladwish, Geordi Pang, Patrick Cheung, Laura D’Alimonte, Andrea Deabreu, Andrew Loblaw

**Affiliations:** Department of Radiation Oncology, University of Toronto, Toronto, Canada; Odette Cancer Centre, Sunnybrook Health Sciences Centre, Toronto, ON Canada; Department of Radiation Oncology, Sunnybrook Health Sciences Centre, 2075 Bayview Ave, Rm T2-103, Toronto, Ontario M4N 3 M5 Canada

**Keywords:** Image-guided radiation therapy, Prostate neoplasms, Intra-fraction motion

## Abstract

**Background:**

To determine prostate displacement during extreme hypofractionated volume modulated arc radiotherapy (VMAT) using pre- and post-treatment orthogonal images with three implanted gold seed fiducial markers.

**Methods:**

A total of 150 image pairs were obtained from 30 patients who underwent extreme hypofractionated radiotherapy to a dose of 40 Gy in five fractions on standard linear accelerators. Position verification was obtained with orthogonal x-rays before and after treatment and were used to determine intra-fraction prostate displacement.

**Results:**

The mean prostate displacements were 0.03 ± 1.23 mm (1SD), 0.18 ± 1.55 mm, and 0.37 ± 1.95 mm in the left-right, superior-inferior, and anterior-posterior directions, respectively. The mean 3D displacement was 2.32 ± 1.55 mm. Only 6 (4%) fractions had a 3D displacement of >5 mm. The average time of treatment delivery for a given fraction was 195 ± 59 seconds.

**Conclusions:**

The mean intra-fraction prostate displacement during a course of extreme hypofractionated radiotherapy delivered via VMAT, continues to be small. Clinical margins typically used in a similar fixed-angle IMRT treatment are adequate. The use of VMAT in further extreme hypofractionation may limit prostatic motion uncertainties that would be otherwise be associated with longer treatment times.

## Background

Prostate cancer is the most commonly diagnosed cancer amongst Canadian men, with approximately 26,000 new diagnoses in 2011 [1]. Radiotherapy remains a mainstain of treatment in a large proportion of these patients. Randomized trials have shown that higher doses of radiotherapy (RT) result in improved biochemical disease free survival, but at the potential cost of increased rectal and urinary toxicity when using traditional 3-dimensional conformal RT (3DCRT) techniques [2,3]. More advanced RT techniques such as intensity-modulated radiotherapy (IMRT) have been shown to be able to deliver these increased doses with less toxicity as compared to 3DCRT [3].

As with any radiotherapy treatment, motion, both intra and inter-fraction is a source of uncertainty. Because of this uncertainty, a margin is added to the clinical target volume (CTV) in creating the planning target volume (PTV). According to ICRU 83, the margin used to create the PTV should account for both internal target motion and day-to-day setup variations [4]. In general, smaller margins may reduce normal tissue toxicity but at the cost of an increased risk of geographical miss [5], while larger margins may maximize tumor control but at the cost of higher normal tissue complications. Accurate characterization of prostate motion may help to determine the optimal margin to maximize complication-free disease-free survival.

Most studies of intra-fraction prostate motion have used conventional 1.8-2 Gy fractions [6,7]. With the discovery of a low alpha-beta ratio for prostate cancer resulting in an increased sensitivity to high dose per fraction [8,9], there has been increased interest in using hypofractionated RT [10,11]. Accurate margins are arguably more pertinent in the hypofractionated setting, as longer treatment times lead to a greater potential for intra-fraction motion and higher doses per fraction mean that geographical misses represent a greater percentage of total dose delivered [6]. This is obviously further accentuated as the degree of hypofractionation increases.

We have previously reported on our experience with prostate motion during extreme hypofractionation (<7 total fractions) based on pre and post-therapy imaging of implanted prostatic fiducial markers [12]. This work was based on conventional fixed-angle RT, which utilizes a planned number of beam angles and achieves intensity modulation via a ‘step-and-shoot’ manipulation of the multi-leaf collimator (MLC). Volume modulated arc therapy (VMAT), which delivers radiation continuously during a 360 degree arc of the linear accelerator while modulating intensity via both fluence output and MLC manipulation, has been shown to reduce treatment times without compromising dose distributions as compared to standard fixed-angle RT in prostate cancer [13]. Our institution, is currently involved in two clinical trials investigating prostate hypofractionated accelerated radiotherapy (pHART), and is utilizing VMAT for treatment delivery. As part of the quality assurance for these studies, portal images of implanted gold seed fiducials were obtained before and after each fraction. This report examines the translational differences in prostate location between these images and compares the results to prior work including both standard and hypofractionated delivery.

## Methods and materials

Patients from this work were drawn from two clinical trials, Prostate Accurately Targeted Radiotherapy Investigation of Overall Treatment Time (PATRIOT) and a phase II study of dose-escalated, Hypofractionated Radiotherapy and Androgen Deprivation Therapy for high-risk prostate cancer (pHART8). Both of these studies were approved by the Sunnybrook Health Sciences Centre Research Ethics Board (REB): REB project identification numbers 385-2011 and 269-2010, respectively. The PATRIOT study randomizes low-intermediate risk prostate cancer patients to 40Gy in 5 fractions delivered either over 29 days (1 fraction per week) or over 11 days (one fraction every other day). The pHART8 study delivers 40Gy in 5 fractions over 29 days (as in arm 1 of PATRIOT) to high-risk prostate cancer patients. Thirty patients were sequentially drawn from the accrual of both trials for analysis in this study. Treatment planning varied slightly between the two trials, as described below, but there were no differences in image acquisition or treatment delivery. Analysis of intra-fraction motion based on fiducial markers was identical in both cases, as was margin estimation.

### Treatment planning

Patients underwent trans-rectal ultrasound-guided insertion of three gold seed fiducials (1 × 3 mm) into the base, mid-gland, and apex of the prostate. Planning computed tomography was performed one week later. Patients were simulated and treated with a comfortably full bladder and empty rectum. No specific medications or diet were given to empty the rectum prior to simulation or treatment. A custom vacuum lock bag was used for immobilization (Vac-Lock, MED-TEC Inc., Orange City, Iowa, USA).

In patients drawn from the PATRIOT trial, the CTV included the prostate only. An isotropic 5-mm margin was added to the CTV to create the PTV. A dose of 40 Gy in five fractions was delivered either once per week over 29 days or every other day over 11 days (as described above). An inverse-planning IMRT technique was used in either case. The Pinnacle v. 9.0 (Philips Radiation Oncology Systems, Fitchburg, WI, USA) software was used for treatment planning.

In patients drawn from the pHART8 trial, there were two clinical target volumes. CTV2 included the prostate only, CTV1 included the inferior 1.5-cm of seminal vesicles or the entire seminal vesicles for patients with cT3b disease. A 5-mm margin was added to each of the CTVs to create PTV1 and PTV2 respectively. An optimized radiotherapy plan was then developed to treat the PTV2 to 40 Gy in 5 fractions; PTV1 received 30 Gy in 5 fractions. One radiotherapy fraction was delivered per week over 29 days. The Pinnacle v. 9.0 (Philips Radiation Oncology Systems, Fitchburg, WI, USA) software was again used for treatment planning.

### Treatment delivery

Patients were treated on linear accelerators (Eleka Synergy, Stockholm, Sweden) equipped with multileaf collimators using VMAT delivery. For each treatment delivery, patients were initially set up according to skin tattoos and in-room lasers. Orthogonal megavoltage electronic portal images of the prostate were then obtained and the implanted fiducial markers were identified and confirmed by two medical radiation therapists. The images were then compared to reference digitally reconstructed radiographs (DRR) from the treatment planning system and translational mismatches were identified using the template matching technique in iViewGT (Eleka, Stockholm, Sweden). The in-house precision of the online targeting and correction process was determined to be 2-mm based on an in-house phantom study, thus only pre-treatment displacements >2 mm were corrected prior to starting treatment [12]. Immediately after treatment, an additional set of orthogonal images was obtained.

### Motion analysis

The minimum prostate motion was calculated as the difference between the pre- and post-treatment images for patients who did not require a couch translation prior to treatment (ie. pre-treatment displacement <2 mm from reference DRR) or the value of the post-treatment shifts alone for patients who underwent correction of displacements prior to treatment. The 3-dimensional (3D) displacement was calculated as the vector using the left-right (LR), superior-inferior (SI), and anterior-posterior (AP) displacements with the formula D = (LR^2^ + SI^2^ + AP^2^)^1/2^.

### Estimation of margin to account for intra-fraction prostate motion

Based on the van Herk formula [14], the CTV-PTV margin needed to cover the CTV with 95% of the dose for 90% of patients is given by:

$$ \mathrm{M} = 2.5\sum + 0.7\sigma $$

Where ∑ is the standard deviation of the systematic error and σ is the standard deviation of the random error.

## Results

### Motion analysis

**Figure 1:**
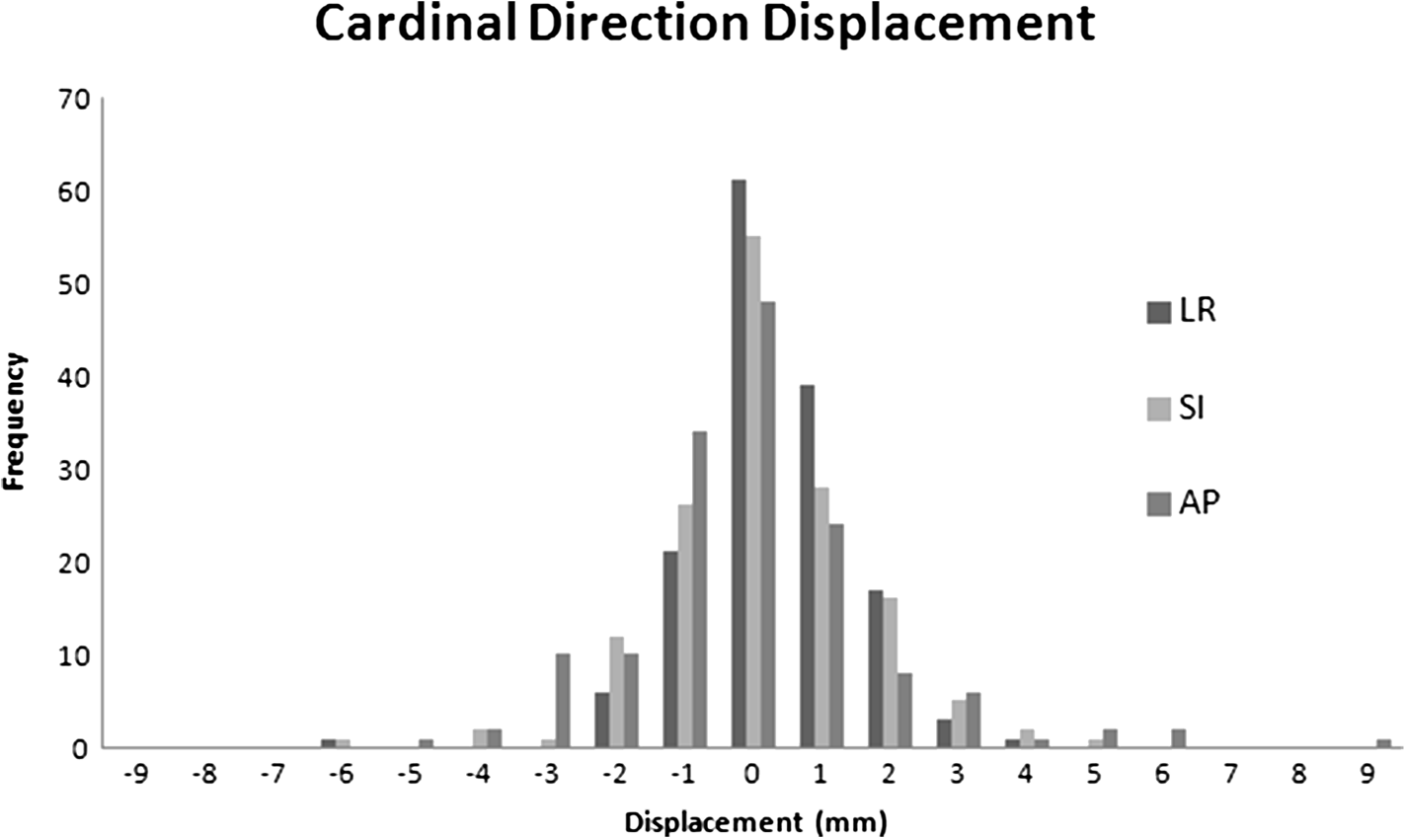
**Frequency and standard deviation of displacements in the Left-right (LR), Superior-Inferior (SI) and Anterior-Posterior (AP) directions.**

A total of 27 patients were taken from the PATRIOT trial and 3 patients were taken from pHART8. A total of 150 sets of pre and post-treatment images were obtained from the 30 patients (five fractions per patient). Of the 150 fractions delivered, 62 (41.3%) exceeded the 2-mm threshold on pre-treatment imaging and required corrective shifting prior to treatment delivery. In general, the displacement of the prostate was small with only 3 (2%) and zero of the total fractions with translations >5 mm and >10 mm in at least one cardinal direction, respectively. The maximum shift seen in any one direction was 8.5 mm, occurring during one fraction in the AP direction. There were two other displacements >5 mm in the AP direction, and none in the LR and SI directions. Each of these occurred in different patients. Displacements were smallest in the LR directions and largest in the AP direction. The mean displacements were 0.02 ± 1.23 mm (1SD) to the right, 0.18 ± 2.30 mm caudally, and -0.37 ± 3.50 mm posterior in the LR, SI, and AP directions, respectively. Figure 1 shows a histogram of the displacements in each of the three cardinal directions.

**Figure 2:**
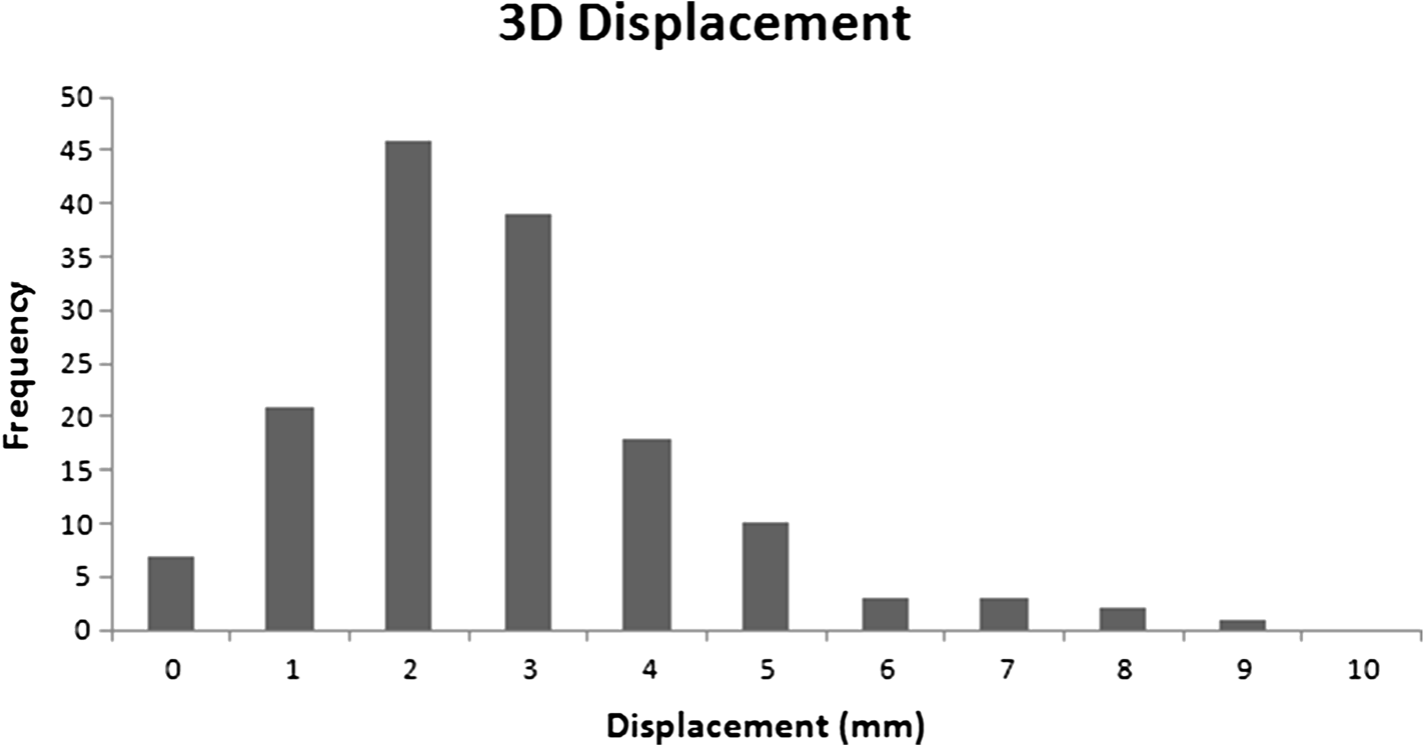
**Frequency of overall displacement in 3-dimensions.**

When analyzing the 3D displacement, 6 (4%) fractions were >5 mm. The maximum 3D displacement was 8.92 mm. The mean 3D displacement was 2.32 ± 1.55 mm. Only a single patient had more than one fraction with >5 mm displacement, and in that patient it was two fractions. A histogram of the 3D displacements is shown in Figure 2.

### Time of delivery

The average time of treatment delivery for a given fraction was 195 ± 59 seconds. The shortest fraction was delivered in 60 seconds. The longest fraction was delivered in 330 seconds.

### CTV-PTV margin

Using the van Herk formula, a margin between the CTV and planning target volume (PTV) was calculated to account for intra-fraction motion. The systematic and random errors in each cardinal direction, as well as the van Herk margin are shown in Table 1. Margins of 2.55 mm, 3.16 mm, and 3.86 mm were calculated in the LR, SI, and AP directions, respectively.

**Table 1:** **Mean (standard deviation) of intrafraction prostate motion**

	**Right-left (mm)**	**Superior-inferior (mm)**	**Anterior-posterior (mm)**
pHART7 and 8 30 patients (8Gy per fraction) VMAT	0.03 (1.23)	0.18 (1.55)	0.37 (1.95)
pHART6 25 patients (8Gy per fraction) IMRT	0.27 (1.26)	0.30 (1.46)	0.79 (1.99)
Previous Study* (7Gy per fraction) IMRT	0.03 (0.61)	0.21 (1.50)	0.86 (1.73)
Previous study** (2 Gy per fraction)	0.14 (0.92)	0.45 (1.27)	0.72 (1.80)

*P. Cheung et al. Int. J. Radiation Oncology Biol. Phys., Vol. 62, pp. 418–425, 2005.

**H. Quon et al. Clinical Oncology, Vol. 24, pp. 640-645, 2012.

## Discussion

Accelerated hypofractionated radiotherapy has been shown to have potential therapeutic benefits in the treatment of prostate cancer. Previous work by Kron et. al. has shown that intra-fraction prostatic motion was related to treatment time [7]. Quon et. al. has shown that despite this, overall prostate displacement remains small and typical organ motion margins utilized typical fixed-angle IMRT margins were adequate for extreme hypofractionation [12]. Tables 1 and 2 show a comparison of VMAT and IMRT for hypofractioned delivery as well as a standard 2Gy fractionation, in terms of treatment motion and corresponding clinical margin (based on the Van Herk formula). Included in these comparisons are the aforementioned study by Quon, utilizing fixed-angle IMRT at 7Gy per fraction (total dose of 35Gy) and a previous in-house study (pHART6) utilizing fixed angle IMRT at 8Gy per fraction (total dose of 40Gy) [15]. Our results are in keeping with previous findings that overall prostatic displacement remains small and that standard clinical margin remain adequate for VMAT hypofractionation.

**Table 2:** **Calculated PTV margin based on Van Herk formula (2.5 SD of systematic error +0.7 SD of random error)**

	**Right-left (mm)**	**Superior-inferior (mm)**	**Anterior-posterior (mm)**
pHART7 and 8 30 patients (VMAT)	2.55	3.16	3.86
pHART6 25 patients (IMRT)	2.29	3.16	4.57
Previous Study* (7Gy per fraction) IMRT	1.40	4.40	5.20

*H. Quon et al. Clinical Oncology, Vol. 24, pp. 640-645, 2012.

The largest experience in dealing with intra-fraction motion in radiotherapy revolves around respiratory changes. Breathing motion is a cyclical pattern and techniques developed to reduce margins in these cases typically involve gating or breath-holding. These are so-called ‘snap-shot’ techniques, treating only with the target is at a certain position, aimed at taking advantage of this reproducible pattern. Intra-fraction prostatic motion is thought to be primarily driven by bladder filling and gaseous rectal emptying/filling, with minor contributions by respiratory motion and other non-specific internal processes [16]. This would present largely as AP and SI displacement, as anatomically the bladder and rectum lie superior/anterior and posterior respectively. Indeed, in this study, AP motion was most prominent, followed by SI motion. This is important, as this pattern is not easily predicable nor reproducible, but rather driven by a continuous process (bladder filling) and a random process (gaseous filling/emptying). Various techniques have been studied to reduce the impact of this motion, including rectal balloons, anti-gas pre-medications and relaxation techniques [17–20]. In theory, shortening treatment times should also be beneficial, as the less time that transpires between setup and treatment completion, the less time there would be for these motion events to occur. Our results indicate that the greatest difference seen between hypofractionated VMAT and fixed angle IMRT is in the AP directions, suggesting that uncertainties associated with random gaseous filling/emptying is reduced with the reduced treatment time.

In this study the average delivery time was just over 3 minutes, roughly half the time of a standard 2Gy fixed-angle IMRT treatment plan and one-third that of an extreme hypofractionated plan [21]. According to Kron, the expected additional displacement of the prostate with relation to treatment time is 0.2 mm/min, or approximately 5-10% additional displacement. We note that in this study, only 6% of fractions had a 3D displacement of >5 mm, as compared to 14% seen in previous work with hypofractionated fixed-angle IMRT. The absolute magnitude of this difference is small, and it did not translate into significant changes in clinical margins by the Van Herk formula, but one can appreciate that as set-up uncertainties decline with improved daily imaging, intra-fraction motion will become increasingly pertinent. If so, volume modulated arc therapy may be useful in reducing overall treatment time and hence organ motion uncertainty.

Limitations of this study include the absence of real-time intra-fraction tracking of prostate motion. Examining the displacement of fiducial markers between pre- and post-treatment imaging only provides a single picture during a dynamic process, and does not capture all potential movement of the prostate. Real-time tracking with implanted via markers has previously been investigated with the Calypso system and more recently with on-board KV imaging [8]. This study did not look to assess pure rotational displacement of the prostate. It is known that rotational motion can be accounted for, in part, by translational displacement, however the relative contribution of each was not assessed in this study. Furthermore, potential deformation of the prostate or displacement of fiducials was not taken into account in this study, however both of these are thought to have minimal impact [22–24]. Finally, this study examined only the geographic changes in the prostate, however the accumulated dosimetric changes and the potential clinical consequences are clearly important aspects [24–26].

## Conclusion

The prostatic displacement over the course of hypofractionated radiotherapy, delivered via VMAT, continues to be small. This suggests that the margins utilized in standard fixed-angle hypofractionated IMRT are adequate. An inherent benefit of VMAT is shorter treatment times, which becomes progressively more significant as the use and degree of hypofractionation increases. A secondary benefit of shortening treatment times may be to limit the organ motion uncertainty that would otherwise be associated with this hypofractionation.
